# Immunomodulatory Activity of Polysaccharide-Protein Complex of Longan (*Dimocarpus longan* Lour.) Pulp

**DOI:** 10.3390/molecules161210324

**Published:** 2011-12-13

**Authors:** Yang Yi, Sen-Tai Liao, Ming-Wei Zhang, Rui-Fen Zhang, Yuan-Yuan Deng, Bao Yang, Zhen-Cheng Wei

**Affiliations:** 1 Key Laboratory of Functional Food, Ministry of Agriculture, Bio-Tech Research Institute, Guangdong Academy of Agricultural Sciences, Guangzhou 510610, China; 2 College of Food Science and Technology, Huazhong Agricultural University, Wuhan 430070, China; 3 South China Botanical Garden, Chinese Academy of Sciences, Guangzhou 510650, China

**Keywords:** longan pulp, polysaccharide-protein complex, immunomodulatory activity, immunosuppressed mice, cyclophosphamide

## Abstract

The immunomodulatory function of longan pulp polysaccharide-protein complex (LP3) was investigated in immunosuppressed mice models. Compared with the model control, peroral administration of 100 mgkg^−1^d^−1^ LP3 could significantly increase/enhance antibody production against chicken red blood cell (CRBC), concanavalin A (ConA)-induced splenocyte proliferation, macrophage phagocytosis, NK cell cytotoxicity against YAC-1 lymphoma cell, and interferon-gamma (INF-γ) and interleukin-2 (IL-2) secretion in serum (*P* < 0.05). The immunomodulatory effects, except for those on splenocytes and macrophages (*P* > 0.05), were also observed in mice administered with 50 or 200 mgkg^−1^d^−1^ LP3 (*P *< 0.05). The beneficial effects of 50–200 mgkg^−1^d^−1^ LP3 were comparable to those of 50 mgkg^−1^d^−1^ ganoderan. The strong immunomodulatory activity of LP3 confirmed its good potential as an immunotherapeutic adjuvant.

## 1. Introduction

Longan (*Dimocarpus longan* Lour.) is widely distributed throughout the Southeast Asia, and its pulp has been used for a long time in folk remedies to promote blood metabolism, soothe nerves, relieve insomnia, and prevent forgetfulness [1,2]. The administration of longan pulp extracts display memory-enhancing, anxiolytic, immunomodulatory, antitumor and antioxidation effects in mice [2,3,4,5], and further analysis points out that the potential active components may be polysaccharides. In fact, botanical polysaccharide-protein complexes, such as those from *Ganoderma lucidum* [6,7,8], *Lycium barbarum* [9,10,11], *Lentinus edodes* [12] and *Agaricus blazei *[13], exhibit a number of therapeutic properties. In addition, our previous work has demonstrated that the polysaccharide-protein complex of longan pulp (LP3) could stimulate splenocyte proliferation, macrophage phagocytosis of neutral red, and NK cell cytotoxicity against YAC-1 lymphoma cell *in vitro* (*P *< 0.05) [14]. However, data concerning its immunomodulatory effect *in vivo*, which is important in explaining the medical application of longan pulp, is still unavailable.

Cyclophosphamide (Cy) is an alkylating agent commercially used in antineoplastic chemotherapy. Both experimental and clinical results have demonstrated an apparently paradoxical effect of Cy on the tumor-host immune response. The better anti-tumor effect of Cy depends on the larger dose of Cy administered. However, along with a reduction of the tumor mass, large doses of Cy usually bring an impairment of the host defense mechanisms, leading to immunosuppressive and cytotoxic effects [15,16]. Polysaccharides from *Ganoderma lucidum*, *Potentilla anserina *and *Sophora subprosrate* have strong immunopromoting effects on Cy-immunosuppressed mice [17,18,19]. To explore the possibility for adjuvant immunotherapy in severely immunosuppressed patients, the immunomodulatory activity of LP3 was evaluated by the model of intensive chemotherapy with Cy in mice, and its effects on cellular and humoral immunity were investigated. In addition, ganoderan, which has been widely proven to up-regulate immune function *in vivo* [19], has been used as a positive control. 

## 2. Results and Discussion

### 2.1. Effect of LP3 on the Production of Serum Hemolysin

**Figure 1:**
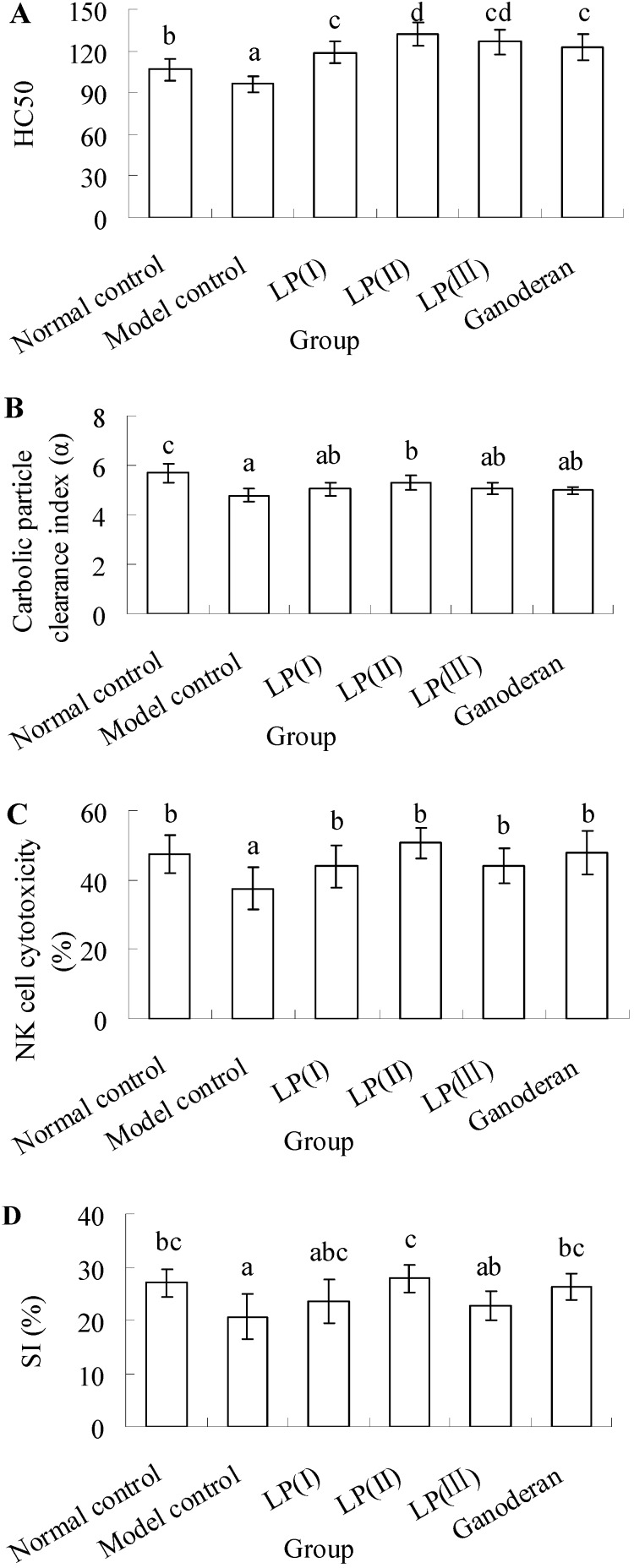
Effects of longan pulp polysaccharide-protein complex LP3 on (**A**) serum hemolysin production; (**B**) macrophages phagocytosis; (**C**) NK cell cytotoxicity; and (**D**) splenic lymphocyte proliferation. Data presented were means ± standard deviation (n = 10). The significant differences among the groups were evaluated with ANOVA followed by the S-N-K test. Columns with different letters are significantly different (*P *< 0.05).

To investigate the effect of LP3 on the immunosuppressed complement system, the content of serum hemolysin in response to cellular antigen was measured. The results shown in Figure 1A indicate that the production of serum hemolysin is noticeably suppressed in the model control compared with the normal (*P *< 0.05). In the LP and ganoderan groups, the hemolysin levels are significantly higher than that of the normal one (*P *< 0.05). The highest upregulation of hemolysin level is found in LP(II), which shows no significant difference in comparison with LP(III) (*P *> 0.05).

### 2.2. Effect of LP3 on the Phagocytosis of Macrophages

As seen in Figure 1B, the carbolic particle clearance index in the model control is significantly lower than that in normal control (*P *< 0.05). Although the index of LP(II) is still lower than the normal (*P *< 0.05), it is obviously improved compared with the model (*P *< 0.05). The differences of carbolic particle clearance among model control, LP(I), LP(III) and ganoderan exhibit no statistical significance (*P *> 0.05).

### 2.3. Effect of LP3 on the Cytotoxicity of NK Cells

Tumor cell elimination is known to be mediated by the cytotoxic activity of NK cells. Therefore, the splenocyte cytotoxicity against NK-sensitive tumor cell (YAC-1) was investigated. As shown in Figure 1C, the NK cell cytotoxicity of model control is visibly suppressed compared with other groups (*P *< 0.05). In LP and ganoderan groups, those responses are maintained at the normal level, which exhibit no significant difference (*P *> 0.05).

### 2.4. Effect of LP3 on the Proliferation of SPL

To confirm the effect of LP3 on the cellular immune response, the proliferation of SPL in response to ConA was evaluated. The results in Figure 1D manifest that the proliferation of SPL in model control is significantly decreased compared with the normal (*P *< 0.05). Nevertheless, 100 mgkg^−1^d^−1^ LP can enhance the proliferation, and its SI is restored to the normal level, as well as 50 mgkg^−1^d^−1^ ganoderan. Both the indices of LP(I) and LP(III) show no significant difference with that of model control (*P *> 0.05).

### 2.5. Effects of LP3 on the Productions of INF-γ and IL-2 in Serum

**Figure 2:**
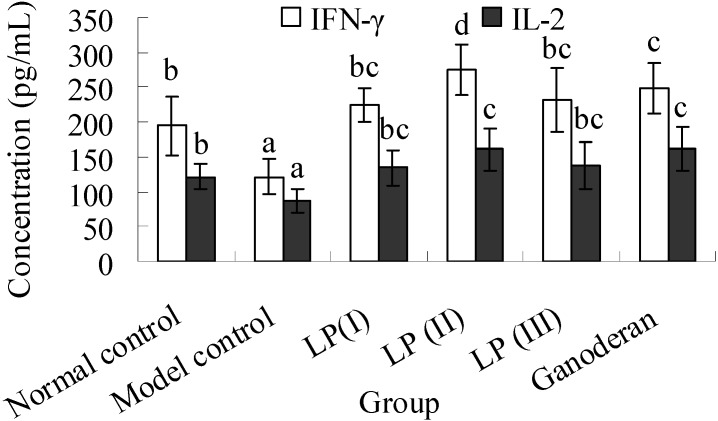
Effect of longan pulp polysaccharide-protein complex LP3 on IFN-γ and IL-2 production. The levels of INF-γ and IL-2 in mice serum were quantified by an ELISA kit. Data presented were means ± standard deviation (n = 10). The significant differences among the groups were evaluated with ANOVA followed by the S-N-K test. Columns with different letters are significantly different (*P *< 0.05).

According to the data shown in Figure 2, the secretions of IFN-γ and IL-2 in the model decrease greatly compared with the normal (*P* < 0.05). The productions of IFN-γ and IL-2 in LP and ganoderan groups are maintained at the normal level. Moreover, the IFN-γ and IL-2 levels in LP(II) and ganoderan groups are upregulated significantly in contrast to that of normal control (*P *< 0.05). Both the levels of IFN-γ and IL-2 among LP(I), LP(III) and ganoderan groups show no significant differences (*P *> 0.05). The serumal IFN-γ concentration of immunosuppressed mice was close to that of Sarcoma 180-bearing mice, but higher serumal IL-2 concentration was measured in immunosuppressed mice. The IFN-γ production up-regulated by LP3 was comparable to the effect of *Lentinus edodes* polysaccharide in tumor-mice [20].

### 2.6. Effects of LP3 on the Indices of Spleen and Thymus

**Figure 3:**
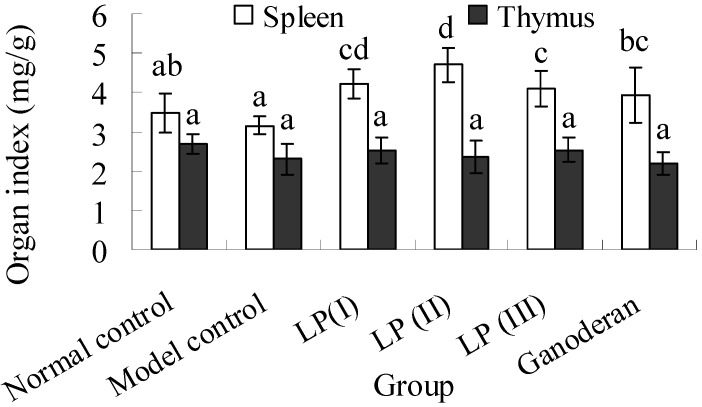
Effect of polysaccharide-protein complex of longan pulp (LP3) on spleen and thymus indices. Spleen/thymus index was measured in the ratio of the spleen/thymus weight (mg) to bodyweight (g). Data presented were expressed as means ± standard deviation (n = 10). The significant differences among the groups were evaluated with ANOVA followed by the S-N-K test. Columns with different letters are significantly different (*P *< 0.05).

As the data in Figure 3 shows, the spleen index in the model control is slightly lower than the normal one. However, no significant difference is found between them (*P *> 0.05). The spleen indices in the LP and ganoderan groups show significant increases compared with that of model control (*P *< 0.05). No significant difference is found on the thymus index (*P *> 0.05). The spleen index range of 3.16–4.70 mg/g was obviously lower than that reported by Chen *et al.* [18]. As an explanation for this, the body weights of Kunming mice in the present work markedly increased after 20 days, but those of BALB/c mice in the previous study were almost constant after a week.

### 2.7. Effects of LP3 on the Body Weight, Water-Intake and Food-Intake

**Figure 4:**
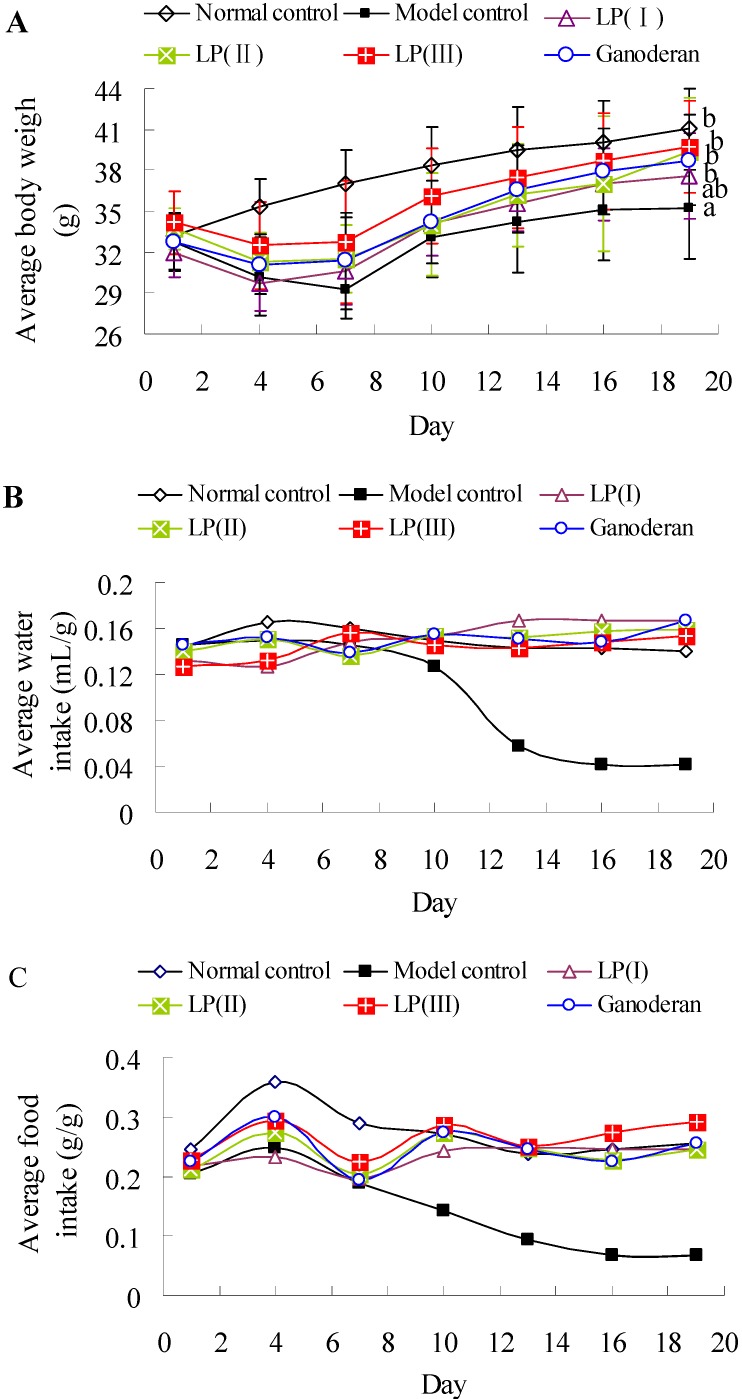
Effects of LP on the bodyweight (**A**); water-intake (**B**); and food-intake (**C**) of immunosuppressed mice. The average bodyweight of each group was expressed as means ± standard deviation (n = 10). The significant differences among the groups were evaluated with ANOVA followed by the S-N-K test. Data points with different letter sinfigure A are significantly different (*P *< 0.05). Average water-intake (mL/g) was calculated as the ratio of total water-intake to total body weight in each group, and average food-intake (g/g) was calculated as the ratio of total food-intake to total body weight.

The body weights of mice were measured to test whether body weight could respond to the restoration of immune system (Figure 4A). Average body weights of all groups exhibit no significant difference at the 1st day (*P *> 0.05). After the intraperitoneal injection of Cy, the bodyweights are decreasing in the immunosuppressed groups during days 4–7, and significant decreases are observed compared with normal control at days 7–10. At the 19th day, the body weight of model control animals is significantly lower than that of other groups except LP(I) (*P *< 0.05). However, the body weights of normal control, LP(I), LP(II), LP(III) and ganoderan groups show no significant difference (*P *> 0.05). As shown in Figure 4B, the average water-intake of model control is obviously decreasing during days 7–16, and those of others exhibit less changes. Each average food-intake is increasing during days 1–4 and then decreasing during days 4–7 (Figure 4C). The average food-intake of model control is gradually decreasing after 4 days. The peroral administration of LP3 could thus effectively improve the body weight and food/water-intake of immunosuppressed mice.

The immunomodulatory functions of aqueous extract and slurry of longan pulp (LE and LS) in normal mice have been reported by Su *et al.* [3], and higher polysaccharide content might contribute to the better effects of LE on serum hemolysin production, spleen lymphocyte proliferation and NK cell activity compared with those of LS. In the present study, LP3 exhibited beneficial actions on the specific and nonspecific immunity of immunosuppressed mice at the optimal dose of 100 mgkg^−1^d^−1^. Likewise, crude polysaccharides from longan pulp could significantly stimulate delayed-type hypersensitivity response, macrophage phagocytosis and splenocyte proliferation in S180 tumor mice [4]. Therefore, it could be postulated that the protein-bound polysaccharides contributed to the immunomodulatory activity of longan pulp. 

Cy has been reported to suppress humoral and cellular immune responses. The actions of Cy are primarily directed toward the depletion of T/B lymphocytes and the deficiency of macrophages [21]. In this work, the humoral and cellular immune responses of immunosuppressed mice were obviously inhibited compared with the normal, and the suppressed responses were consistent with the previous studies [18,19,21,22]. Several visual symptoms, such as lethargy, alopecia, unusual weakness and anorexia were observed as described by Ma *et al.* [23]. After the Cy treated mice received intraperitoneal injection of 2.5 or 25 mgkg^−1^d^−1^ ganoderan once daily for eight days, recoveries of lymphocyte proliferation, macrophage phagocytosis, splenic NK cell cytotoxicity were observed [19], and these functions of ganoderan were also confirmed by our results. In addition, the activities of lymphocyte, macrophage and NK cell of immunosuppressed mice were significantly up-regulated by the peroral administration of 100 mgkg^−1^d^−1^ LP3 for 15 days, and the recoveries of bodyweight and food-intake were observed after seven days (Figure 4A and 4C). The results demonstrated that peroral administration of LP3 afforded a beneficial regulation on humoral and cellular responses against Cy-induced immunosuppressed effects, and implied the potential application of LP3 as a natural immunomodulating agent.

Immunization with CRBC results in the secretion of specific antibody in serum, which can lead to the cytolytic response against CRBC. It was reported that both degraded glucan and native polysaccharides from *Ganoderma lucidum* could obviously increase the production of antibodies against sheep red blood cells in mice [24]. LP3 and ganoderan stimulated the production of antibody against CRBC *in vivo* (Figure 1A), which implied the promotion of humoral immunity by stimulating antibody-secreting B-cells in spleen. Meanwhile, the therapy with LP3 might cause the compensation of T-cell deficiency (which induced the recovery of spleen and the stimulation of lymphocyte) and the stimulations of macrophage and NK cell (Figure 1B-D). The increased levels of IL-2 and IFN-γ in the sera of immunosuppressed mice suggested that LP3 not only induced a restoration of decreased T-cell-dependent antibody response by the enhancement of IL-2-production and but also stimulated macrophage phagocytosis by the enhancement of IFN-γ production. Moreover, Th1 cells were the potentially target of LP3 [25].

## 3. Experimental

### 3.1. Materials and Chemicals

Fresh fruits of longan (cv. Chu-liang) at the mature stage were provided by the Pomology Research Institute of Guangdong Academy of Agricultural Sciences (Guangzhou, China). Cy was purchased from Heng Rui Medicine CO., Ltd. (Jiangsu, China). India ink was obtained from Chroma-Gesellschaft (Köngen, Germany). 3-[4,5-Dimethylthiazol-2-yl]-2,5-diphenyltetrazolium bromide (MTT) and concanavalin A (ConA) were purchased from Sigma (St. Louis, MO, USA). Mouse INF-γ and IL-2 ELISA kits were obtained from Neobioscience Technology Co., Ltd. (Shenzhou, China). RPMI-1640 medium (Grand Island, NY, USA) was supplemented with 10% fetal bovine serum (Gibco BRL) as complete medium. Ganoderan was provided by Orient Plant Health Care Sci. and Tech. CO., Ltd. (Huizhou, China), and 95.2% polysaccharide content was measured by phenol-sulphuric acid method [26].

### 3.2. Preparation of LP3

The prior treatment of longan fruits and the preparation of LP3 were implemented according to our previous methods [14]. In brief, crude polysaccharides in dried longan pulp were extracted with distilled water using cellulase enzymolysis and ultrasonic cell disintegration. The polysaccharide extract was then purified by the static absorption of anion exchange resin D301-F (Jiangsu Suqing Water Treatment Engineering Group Co., Ltd., Jiangyin, China) to remove pigments and proteins. The purified polysaccharide solution was dialyzed against distilled water followed by centrifugation. The obtained supernatant was concentrated and finally lyophilized as polysaccharide-protein complex LP3. Its ratio of carbohydrate to protein was 30:1. The carbohydrate portion was composed of ribose, rhamnose, arabinose, xylose, mannose, glucose and galactose in the molar ratio of 4.85:1.06:14.55:1.00:28.36:70.89:8.58. Sixteen amino acids were measured in LP3 with the hydrophobic amino acid/protein ratios of 1.00:2.81 [14].

### 3.3. Animals and Cells

Specific pathogen-free Kunming mice (male, 20.0 ± 2.0 g, certificate number: SCXK-Yue 2006-0015) were purchased from Laboratory Animal Sciences Center of Southern Medical University (Guangzhou, China). The mice were bred on a 12-h-dark/12-h-light cycle at 22 ± 2 °C and allowed free access to standard laboratory rodent diet (Laboratory Animal Sciences Center of Southern Medical University, China) and tap water. The animals were allowed to adapt to the environment for one week before the experiments. Three batches of mice were bred for the whole investigation, and each of which was composed of six groups (ten mice per group). Groups with different treatments *in* *vivo* experiments were showed in Table 1. 

**Table 1:** Groups of mice with different treatments *in vivo* experiments.

**Groups**	**NSS (mLkg^−1^d^−1^)**	**Cy (mgkg^−1^d^−1^)**	**NSS (mLkg^−1^d^−1^)**	**LP3 (mgkg^−1^d^−1^)**	**Ganoderan (mgkg^−1^d^−1^)**
	Intraperitoneal injection (days 1–3)		Peroral administration (days 4–18)		
Normal control	5	–	5	–	–
Model control	–	80	5	–	–
LP(I)	–	80	–	50	–
LP(II)	–	80	–	100	–
LP(III)	–	80	–	200	–
Ganoderan	–	80	–	–	50

NSS: normal sodium solution; Cy: cyclophosphamide; LP3: longan pulp polysaccharide-protein complex 3.

The dose of ganoderan referred to the report of Bao *et al.* [27]. The tests were performed on day 19 and were approved by the Laboratory Animal Committee of Guangdong Province. All the treatments to animals were performed in accordance to the Guide for the Care and Use of Laboratory Animals. YAC-1 lymphoma cell line was provided by Experiment Animal Center of Sun Yat-sen University (Guangzhou, China).

### 3.4. Measurement of Serum Hemolysin

Seven days before the last administration, each mouse was intraperitoneally injected with 0.2 mL 5% (v/v) CRBC. Mice were sacrificed by blood letting via the eyeball, and the blood of each animal was collected into a 1.5 mL Eppendorf tube and centrifuged at 2,500 rpm for 20 min to isolate serum. The serum was 150 times diluted by normal saline solution (NSS). Diluted serum (1 mL) was then incubated with 2% CRBC (0.5 mL) and 10% guinea pig serum (0.5 mL) in a 2 mL sterile tube at 37 °C for 30 min. The response was ended by ice bath. Diluted serum was replaced by NSS in the control. The mixture was centrifuged at 1,800 rpm for 5 min, and the optical density (OD) value of the supernatant was measured at 540 nm. Likewise, 2% CRBC (0.25 mL) and purified water (1.75 mL) were incubated and measured as 50% haemolytic complement (HC_50_) of CRBC. The antibody production against CRBC in serum was expressed by HC_50_ and calculated as: OD_sample_/OD_CRBC_ × n, in which OD_sample_ and OD_CRBC_ respectively represented the OD value of sample and CRBC, and n represented the diluted rate of serum [28].

### 3.5. Phagocytosis Assay of Macrophage

The phagocytosis of macrophages in liver and spleen was assessed by the test of carbon particles clearance [28]. India ink was diluted five times by NSS and then injected via the vena caudalis at the dose of 5 mL/kg. Blood (20 μL) was collected via the eye orbit at 2 min (t_1_) and 10 min (t_2_) after injection, and was immediately mixed with 0.1% (w/v) Na_2_CO_3_ (2 mL) to determine OD_1_ and OD_2 _values at 680 nm, respectively. In addition, the liver weights and spleen weights of the mice were isolated and measured after cervical dislocation. The index of carbolic particle clearance (α) was expressed by the following equation:

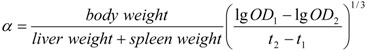


### 3.6. Cytotoxicity Assay of NK Cell

Each mouse was sacrificed by blood letting via eyeball, and the blood was collected into a 1.5 mL Eppendorf tube and centrifuged at 2,500 rpm for 20 min to obtain serum for the determination of cytokine concentration. The spleen was removed and placed into a 1.5 mL Eppendorf tube to be weighed under aseptic condition, and then minced in aseptic phosphate-buffered saline (PBS) through a sterilized mesh (200 mesh) to obtain the single cell suspension. After the cell suspension was centrifuged at 1,000 rpm for 5 min, the supernatant was removed. The remaining cells were mixed with aseptic distilled water (1 mL) for 30 s to deplete red blood cells, and then 1.8% (w/v) NaCl (1 mL) was added in to ensure isosmotic conditions. The cells were washed three times and resuspended to a final concentration of 1 × 10^7^ cells/mL by complete medium. NK-sensitive YAC-1 lymphoma cells were used as target cells and adjusted to 5 × 10^5^ cells/mL by complete medium. The experimental well in a 96-well culture plate was plated with 50 μL splenocytes and 50 μL target cells. The well plated with 50 μL splenocytes was effector control, and that plated with 50 μL target cells was target control. After the incubation of 4 h (37 °C, 5% CO_2_), each well was pulsed with MTT (20 μL, 5 mg/mL) and further incubated for 4 h. Acidified isopropyl alcohol (100 μL) was then added and kept for 12 h to dissolve the formazan crystals. The plate was analyzed at 570 nm using a microplate reader (Thermo Labsytems, Helsinki, Finland). The NK cell cytotoxicity (%) was calculated as: [OD_T_ − (OD_exp_ − OD_E_)]/OD_T_ × 100, in which OD_exp_, OD_E_ and OD_T_ represented the OD values of experimental group, effector control and target control, respectively [29].

### 3.7. Proliferation Assay of Splenic Lymphocyte

The isolated splenocytes from section 3.6 were adjusted to 5 × 10^6^ cells/mL, and 100 μL per well was plated in a 96-well culture plate. The cells were incubated with or without 5.0 μg/mL ConA for 48 h (37 °C, 5% CO_2_) followed by another 4 h with 20 μL MTT (5 mg/mL). Then, each well was pulsed with acidified isopropyl alcohol (100 μL) and measured at 570 nm. The proliferation of splenic lymphocyte (SPL) was expressed by stimulation index, *i.e.*, SI (%) = (OD_stim_ − OD_control_)/OD_stim_ × 100, in which OD_stim_ and OD_control_ represented the OD values of stimulated group and control group, respectively [28,30].

### 3.8. Determination of INF-γ and IL-2 in Serum

The concentrations of INF-γ and IL-2 in the sera from Section 3.6 were determined using ELISA kits according to the instruction of the manufacturer.

### 3.9. Measurement of Spleen and Thymus Indices

The spleen weights and thymus weights of mice from Section 3.6 were measured. The indices of spleen and thymus were calculated as the ratio of organ to body weight.

### 3.10. Measurement of Body Weight, Water-Intake and Food-Intake

Each mouse was weighed daily, while the total water-intake and food-intake of each group were measured. Average water-intake (mL/g) was calculated as the ratio of total water-intake to total body weight, and average food-intake (g/g) was calculated as the ratio of total food-intake to total body weight.

### 3.11. Statistical Analysis

Data was expressed as means ± standard deviation (SD). Significance of difference was evaluated with one way analysis of variance (ANOVA) followed by the Student-Newman-Keuls (S-N-K) test by the SPSS 11.5 software. *P*-values less than 0.05 were used as the threshold for significance.

## 4. Conclusions

The health benefits of longan pulp are partly related to the immunomodulatory activities of polysaccharides. Peroral administration of 100 mgkg^−1^d^−1^ LP3 can significantly stimulate lymphocyte/macrophage activation and cytokine/antibody secretion. LP3 exhibits great potential in clinical application compared with ganoderan. However, the structural characterization and structure/immunomodulation relationship of LP3 should be further investigated in the future.
